# The estrogen receptor-α A908G (K303R) mutation occurs at a low frequency in invasive breast tumors: results from a population-based study

**DOI:** 10.1186/bcr1315

**Published:** 2005-09-06

**Authors:** Kathleen Conway, Eloise Parrish, Sharon N Edmiston, Dawn Tolbert, Chiu-Kit Tse, Joseph Geradts, Chad A Livasy, Harsharan Singh, Beth Newman, Robert C Millikan

**Affiliations:** 1 Department of Epidemiology, School of Public Health, University of North Carolina, Chapel Hill, NC, USA; 2 Lineberger Comprehensive Cancer Center, School of Medicine, University of North Carolina, Chapel Hill, NC, USA; 3 Department of Pathology, Duke University Medical Center, Durham, NC, USA; 4 Department of Pathology and Laboratory Medicine, School of Medicine, University of North Carolina, Chapel Hill, NC, USA; 5 School of Public Health, Queensland University of Technology, Brisbane, Australia

## Abstract

**Introduction:**

Evidence suggests that alterations in estrogen signaling pathways, including estrogen receptor-α (ER-α), occur during breast cancer development. A point mutation in ER-α (nucleotide A908G), producing an amino acid change from lysine to arginine at codon 303 (K303R) results in receptor hypersensitivity to estrogen. This mutation was initially reported in one-third of hyperplastic benign breast lesions, although several recent studies failed to detect it in benign or malignant breast tissues.

**Methods:**

We screened 653 microdissected, newly diagnosed invasive breast tumors from patients in the Carolina Breast Cancer Study, a population-based case-control study of breast cancer in African American and white women in North Carolina, for the presence of the ER-α A908G mutation by using single-strand conformational polymorphism (SSCP) analysis and ^33^P-cycle sequencing.

**Results:**

We detected the ER-α A908G mutation in 37 of 653 (5.7%) breast tumors. The absence of this mutation in germline DNA confirmed it to be somatic. Three tumors exhibited only the mutant G base at nucleotide 908 on sequencing, indicating that the wild-type ER-α allele had been lost. The ER-α A908G mutation was found more frequently in higher-grade breast tumors (odds ratio (OR) 2.83; 95% confidence interval (CI) 1.09 to 7.34, grade II compared with grade I), and in mixed lobular/ductal tumors (OR 2.10; 95% CI 0.86 to 5.12) compared with ductal carcinomas, although the latter finding was not statistically significant.

**Conclusion:**

This population-based study, the largest so far to screen for the ER-α A908G mutation in breast cancer, confirms the presence of the mutant in invasive breast tumors. The mutation was associated with higher tumor grade and mixed lobular/ductal breast tumor histology.

## Introduction

The principal risk factors for breast cancer are hormonal or reproductive factors that increase exposure to estrogen [1]. The importance of estrogen in breast cancer development is further supported by studies demonstrating the occurrence of marked changes in estrogen signaling and expression of the two estrogen receptors (ERs) ER-α and ER-β during breast tumorigenesis and progression [2-8]. Although mutations in the ER-α gene are relatively rare in primary breast cancers [2,3], Fuqua and colleagues recently described a point mutation in ER-α in one-third of typical breast hyperplasias [9], and also observed this mutation in a high percentage of breast tumors [10]. This A→G base substitution at nucleotide 908 in codon 303, referred to as ER-α A908G or K303R, results in an amino acid change of lysine to arginine. The mutation affects the border of the hinge and the hormone-binding domains of ER-α and has been reported to confer hypersensitivity to estrogen compared with wild-type ER-α, leading to increased cellular proliferation at sub-physiologic levels of estrogen [9]. No difference in estradiol affinity was detected between the mutant and wild-type ER-α; however, the mutant exhibited enhanced binding to the TIF-2 coactivator at low hormone levels [9]. Recent studies also indicate that the ER-α A908G or K303R mutation renders the receptor hypersensitive to phosphorylation at Ser305 through the phosphatidylinositol 3-kinase/Akt signaling cascade [11], protein kinase A [12] and p21-activated kinase [10].

The enhanced function of the hypersensitive ER-α A908G mutant and its discovery in early hyperplastic breast lesions makes it a potentially important marker for studies of breast cancer etiology and progression. In the present study, we screened a series of newly diagnosed invasive breast tumors from patients enrolled in the Carolina Breast Cancer Study (CBCS), a population-based case-control study of breast cancer in African American and white women in North Carolina, for the A908G point mutation in ER-α by using a combination of single-strand conformational polymorphism (SSCP) analysis and ^33^P-cycle DNA sequencing. Our results extend the initial observations of Fuqua and coworkers [9,10] by confirming the presence of this mutation in some invasive breast carcinomas.

## Materials and methods

### Study population

The CBCS is a population-based case-control study of breast cancer. Participants include women, aged 20 to 74 years, residing in 24 contiguous counties of central and eastern North Carolina [13]. Women with a first diagnosis of invasive breast cancer between 1993 and 1996 were identified by the North Carolina Central Cancer Registry through a rapid case ascertainment system. Women diagnosed before the age of 50 years and African American women were oversampled to ensure that they comprised roughly half the study sample. Additional details of the study design are described elsewhere [13]. All aspects of this research were approved by the University of North Carolina (UNC) School of Medicine Institutional Review Board. A total of 861 breast cancer cases were eligible for and consented to participate in the CBCS. Epidemiologic risk factor information was obtained from questionnaires that were administered to participants in their homes by trained nurse-interviewers. Clinical data and information on tumor characteristics were obtained from medical records or by a direct histopathologic review of tumor tissue. The ER status of breast tumors was determined primarily through a review of medical records (*n *= 567), and by immunohistochemical staining in the remaining cases (*n *= 62) in the Tissue Procurement and Analysis Facility at UNC as described previously [14].

### Tumor tissue preparation and histopathologic evaluation

Formalin-fixed paraffin-embedded tumor blocks were obtained from pathology departments at participating hospitals for 798 of the 861 breast cancer cases. Of these, 684 had sufficient tumor tissue for molecular analyses. Tumors were sectioned as described previously [15] and underwent standardized histopathologic review by the study pathologist (JG), which included scoring of each tumor for histologic grade, nuclear grade and mitotic index. These three features were considered in assigning the Nottingham score (of 1 to 9), which was then transformed to a three-level combined grade (grades 1 to 3).

With the hematoxylin/eosin-stained slide as a guide, the area of tumor was microdissected away from other surrounding non-tumor tissue, and DNA lysates were prepared for molecular analyses by using Proteinase K extraction.

Of the 684 tumors available for molecular studies, 653 were successfully screened for mutations in a 104-bp region of exon 4 surrounding codon 303 of ER-α, using a combination of SSCP and ^33^P-cycle DNA sequencing. The tumors that were screened for ER mutations were more likely to be of later stage (*P *= 0.005), larger size (*P *= 0.0002), lymph node positive (*P *= 0.006), and higher combined grade (*P *= 0.04) than those that were not screened, which is consistent with the greater availability of tumor tissue from larger breast tumors. However, the cases screened for mutations did not differ from those that were not screened on age (*P *= 0.42), menopausal status (*P *= 0.90), race (*P *= 0.63), ER status (*P *= 0.68), or breast tumor histologic subtype (*P *= 0.48).

### PCR amplification of ER-α exon 4

A 104-bp fragment of exon 4 surrounding codon 303 was amplified with primers ER4A (5'-ATGAGAGCTGCCAACCTT-3') and ER4BS (5'-AACAAGGCACTGACCATCT-3'). Reactions were performed in 1 × PCR buffer (50 mM KCl, 10 mM Tris-HCl, pH 8.3, 1.5 mM MgCl_2_, 0.001% gelatin), with 100 μM each of the four deoxyribonucleotide triphosphates, 1.25 units of AmpliTaq Gold DNA Polymerase (ABI), 0.6 μM of each primer, and 1 μl DNA lysate under the following cycle conditions: one cycle of 95°C for 8 min, 35 cycles of 95°C for 1 min, 60°C for 1 min and 72°C for 1 min, and a final extension at 72°C for 10 min. Handling of all tissues and DNA and the performance of initial PCR reactions were performed in a separate clean room to avoid contamination by PCR products.

### SSCP screening

Mutations within the 104-bp region of ER-α exon 4 surrounding codon 303 were evaluated by SSCP analysis. First-round PCR product was diluted in distilled H_2_O (about 1:25) and 1 μl was used in a 20 μl SSCP-PCR reaction containing each primer ER4A and ER4BS at 600 nM, 1 × PCR buffer, each dNTP at 150 μM (except 22.5 μM dCTP and 0.2 μl of α-labeled ^32^P-dCTP (ICN)), and 0.5 unit of AmpliTaq Gold DNA Polymerase (ABI). Cycling parameters were one cycle of 95°C for 5 min, 60°C for 1 min and 72°C for 1 min, 33 cycles of 94°C for 1 min, 60°C for 1 min and 72°C for 1 min, and a final extension of 94°C for 1 min followed by 60°C for 10 min. The SSCP-amplified PCR product was diluted 1:50 in 0.1% SDS and 10 mM EDTA, mixed with 92% formamide and 40 mM EDTA stop dye at a 1:1 ratio, denatured, and analyzed by PAGE on a 6% (5.8:0.2 acrylamide : bisacrylamide ratio) polyacrylamide gel containing 1 × Tris/borate/EDTA buffer along with positive, negative, and undenatured control samples. Gels were run at 40 W at 4°C and transferred to chromatography paper, dried, and then exposed to film (Hyperfilm MP) at -80°C for 2 days. SSCP was repeated on at least 10% of tumors that initially gave a wild-type SSCP result (*n *= 69), and all of these tumors again showed only the wild-type SSCP pattern.

### ^33^P-cycle sequencing

Tumor samples exhibiting abnormal band migration by SSCP were sequenced on both the forward and reverse DNA strands by using ^33^P-labeled cycle sequencing methods. The PCR products were incubated at 37°C with ExoSAP-IT (USB) (2 μl per 5 μl PCR product) for 15 min before sequencing. Cycle sequencing was performed with the Thermo Sequenase Radiolabeled Terminator Cycle Sequencing Kit (USB) in accordance with the manufacturer's instructions, using either primer ER4A or ER4BS for 30 cycles of 95°C for 30 s, 62°C for 30 s, and 72°C for 1 min. Stop solution (95% formamide, 20 mM EDTA, 0.05% bromophenol blue, 0.05% xylene cyanol FF) was added and samples were heated to 70°C for 5 to 10 min before being run on an 8% polyacrylamide standard sequencing gel. Gels were dried on chromatography paper and then exposed to film (Hyperfilm MP). All mutations were confirmed (and the possibility of mutation artifacts was ruled out) by sequencing of a second, separately amplified PCR product. Additionally, at least 5% of SSCP-negative tumors were sequenced (*n *= 46), but no mutations were found in these samples.

### ^35^S manual sequencing

The ER-α exon 4-amplified 104-bp PCR products were incubated at 37°C with ExoSAP-IT (USB) (2 μl per 5 μl of PCR product) for 15 min before asymmetric PCR. Asymmetric PCR reactions were prepared to generate single-stranded DNA products in both the forward and reverse directions. The forward asymmetric PCR reaction consisted of 300 nM primer ER4A and 6 nM primer ER4BS; the reverse reaction contained 6 nM primer ER4A and 300 nM primer ER4BS. All other reaction conditions were the same as described for first-round PCR. The amplified single-stranded products were prepared for sequencing by filtration through Centricon 30 spin filters (Amicon). The products were sequenced with the Sequenase 2.0 dideoxy-termination method with the use of ^35^S-dATP to reveal the bands. All mutations were confirmed by sequencing in a separately amplified aliquot of DNA to rule out mutation artifacts.

### Automated fluorescent sequencing

Sequencing was conducted at the UNC DNA Sequencing Core Facility on a 219-bp PCR product amplified from ER-α exon 4 with primers ER5'#1 (5'-AACACAAGCGCCAGAGAG-3') and ER4B (5'-CTGAAGGGTCTGGTAGGA-3'). The PCR product was purified by using ExoSAP-IT (USB; 2 μl per 5 μl of product), and was cycle sequenced with fluorescently labeled Big Dye v1.1 terminators (ABI) on a 3730 DNA Analyzer (ABI) with a 48-capillary array. Foundation Data Collection v2 (ABI) software was used to collect and analyze the sequencing data.

### SNaPshot dideoxy primer extension assay

ER-α exon 4 PCR-amplified products were incubated at 37°C with ExoSAP-IT (USB; 2 μl per 5 μl of PCR product) for 15 min before mutation screening with SNaPshot (ABI). SNaPshot was performed with a 1:10 (about 0.2 pM) dilution of the purified ER-α exon 4 PCR-amplified product with primers ERSNP303-5' (5'-CGCTCATGATCAAACGCTCTAAGA-3') at 1.0 μM final concentration and ERSNP303-3' (5'-AAGGCCAGGCTGTTC-3') at 2.0 μM final concentration in the SNaPshot Multiplex Ready Reaction Mix (ABI) with the following cycle conditions: 96°C for 20 s, 64°C for 10 s, 72°C for 30 s, for 25 cycles. The reactions were then treated with 1 unit of shrimp alkaline phosphatase (Promega) and incubated for 1 hour at 37°C followed by deactivation at 75°C for 15 min. The samples and SNaPshot controls were analyzed on the ABI 377 Genetic Analyzer with the GeneScan data analysis program (ABI).

### Positive control

A formalin-fixed paraffin-embedded non-study hyperplastic benign breast tissue was confirmed by ^33^P and ^35^S sequencing to carry the ER-α A908G mutation and this sample was used as a positive control throughout the screening studies. This tissue also produced a prominent band shift on SSCP and was positive for the mutation by SNaPshot. Mutant ER-α exon 4 PCR product was cloned from this control sample and several clones were sequenced for further confirmation of the presence of the mutation in this tissue.

### Statistical analysis

ER-α variants were evaluated for prevalence and type. Using SAS software (SAS Institute, Cary, NC), χ^2 ^statistics and odds ratios (ORs) calculated using logistic regression were used to measure the association between the ER-α A908G mutation and clinical or other characteristics. All *P *values were two-sided.

## Results

### Detection of the ER-α A908G mutation and other sequence variants

The demographic and clinical characteristics of the breast cancer cases evaluated for the ER mutation are given in Table 1. Slightly more than half (51.1%) of the 653 cases were premenopausal and 39.1% were African American. Most cases had early American Joint Committee on Cancer stage 1 or 2 breast cancer (88.3%) and were lymph-node negative (60.5%).

**Figure 1:**
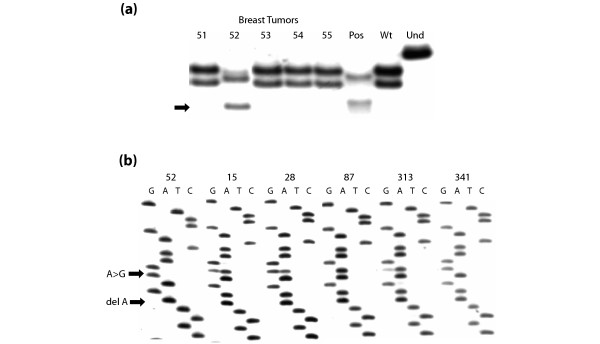
SSCP and sequencing analysis of the ER-α A908G mutation in invasive breast tumors. **(a) **Single-strand conformational polymorphism (SSCP) analysis showing the band shift characteristic of the estrogen receptor-α (ER-α) A908G mutation in ductal breast tumor 52 and in the positive control tissue MN31 (Pos). Tumors 51 and 53 to 55 show the wild-type SSCP pattern, as does the MCF-7 negative control cell line (Wt). Und is the undenatured wild-type control. **(b) **^33^P-Cycle sequencing analysis confirming the presence of the ER-α A908G mutation in breast tumors 52, 15, 28, 313 and 341, as indicated by the upper arrow (A→G). Tumor 87 shows only wild-type ER-α. Note that tumor 52 also carried a second mutation, a deletion of A in codon 302, indicated by the lower arrow (del A), which seems to be in the allele exhibiting the A908G mutation in codon 303.

The ER-α A908G mutation was detected in 37 of 653 (5.7%) invasive breast tumors. Tumor samples carrying the A908G mutation produced a characteristic band shift on SSCP, similar to that exhibited by the positive control benign breast tissue, as shown in Fig. 1a. DNA sequencing confirmed the presence of the mutation in tumors exhibiting the variant SSCP pattern, including tumor 52, as shown in Fig. 1b. Some tumors showed intense mutant ER-α A908G bands on sequencing (tumors 15, 28, 52, and 341), whereas others showed very faint bands (tumor 313), suggesting the presence of this mutation in only a minor subpopulation of cells in some tumor tissues. Three tumors, including tumors 52 and 341 (Fig. 1b), exhibited only the mutant base G at position 908 on sequencing, indicating that these tumors had undergone loss of the wild-type ER-α allele.

To confirm the somatic nature of the ER-α A908G mutation, we sequenced germline DNA extracted from the peripheral blood of 27 of the 37 positive CBCS cases, including all those whose breast tumors prominently displayed the mutation, because these cases would be most likely to carry a germline change. The A908G base change was not detected in the germline of any case.

In addition to carrying the A908G mutation in codon 303, tumor 52 also exhibited a one-base deletion (of A) in codon 302, resulting in a frame shift that would be expected to produce a null ER-α protein. The two mutations probably occurred on the same allele because only the double-mutant sequence was observed; the wild-type sequence was absent from this tumor (Fig. 1b). Neither mutation was detected in corresponding germline DNA from this case. Tumor 52 was negative for ER expression, which would be consistent with production of only the truncated ER-α protein.

The previously reported AC**G**→AC**A **silent variant in codon 311 [16] was identified in three tumors by SSCP and sequencing, and its presence was also confirmed by sequencing the corresponding germline DNA. All three cases carrying the codon 311 polymorphism were African American, which is consistent with the study of Schubert and colleagues [16], which evaluated 105 cases and 151 controls from the CBCS. However, the codon 311 variant did not seem to be linked with a codon 309 (T**C**C→T**T**C) polymorphism also described in their report, because we did not detect the codon 309 variant in any CBCS cases by SSCP and sequencing, including those who carried the codon 311 polymorphism.

### Clinical and patient characteristics associated with the ER-α A908G mutation

We evaluated the association of the ER-α A908G mutation with demographic and clinical characteristics of the breast cancer cases. The mutation was detected in 4.5% of premenopausal and 6.9% of postmenopausal cases, whereas 6.3% of white cases and 4.7% of African American cases carried the mutation. These differences were not statistically significant (Table 1). Cases who were older than 50 years of age and whose tumors were later stage, of larger size, and lymph-node positive were somewhat more likely to have mutation-positive breast cancers, although these results were not statistically significant. In particular, breast tumors having a higher combined grade were more likely to carry the ER-α A908G mutation (odds ratio (OR) = 2.83, 95% confidence interval (CI) = 1.09 to 7.34 for grade II; OR = 1.65, 95% CI = 0.60 to 4.59 for grade III compared with grade I). There was a weak positive, but not statistically significant, association between ER-α A908G mutation status and ER protein expression in breast tumors. However, because most ER data were abstracted from medical records and detailed quantitative data on the percentage tumor cells expressing ER were not available [14], the precise relationship between ER-expressing cells and those carrying the mutation could not be determined.

Breast tumors in the CBCS were classified histologically into four groups. The prevalence of the ER-α A908G mutation was 11.5% in mixed lobular/ductal tumors, 5.3% in ductal NOS (not otherwise specified), 4.8% in ductal variants, and 3.4% in lobular tumors. Mixed lobular/ductal tumors were more likely than ductal tumors to carry the ER-α A908G mutation (OR = 2.10, 95% CI = 0.86 to 5.12), although this result was not statistically significant (Table 1). It should be noted that the initial histopathologic review of these CBCS tumors took place in the mid-1990s; however, according to more updated histopathologic criteria, several of the seven mutation-positive mixed lobular/ductal tumors would now be described as 'ductal carcinoma with lobular features'. Only one ER-α A908G mutation-positive variant lobular tumor was considered possibly a pleomorphic lobular carcinoma, but information on E-cadherin expression was not available for this tumor to confirm this diagnosis. The higher prevalence of the A908G mutation in mixed lobular/ductal tumors might explain in part the stronger association of the mutation with tumor grade II rather than grade III, because breast tumors with a lobular component tend to be of lower grade than non-lobular tumors.

### Comparison of ^33^P-cycle sequencing with methods used in previous studies

Since the initial discovery of the ER-α A908G mutation in breast hyperplasias [9], four studies have failed to detect this variant in benign or malignant breast tissues [17-20]. Three of these used fluorescent DNA sequencing [18-20] and one used a *Mbo*II restriction digest approach [17]. To determine whether differences in laboratory methods contributed to these negative results, we performed several direct comparisons between ^33^P-cycle sequencing, which we used to confirm SSCP-positive findings in the CBCS, and fluorescent DNA sequencing with Big Dye terminators, ^35^S manual DNA sequencing, and the SNaPshot dideoxy single-base extension method.

**Figure 2:**
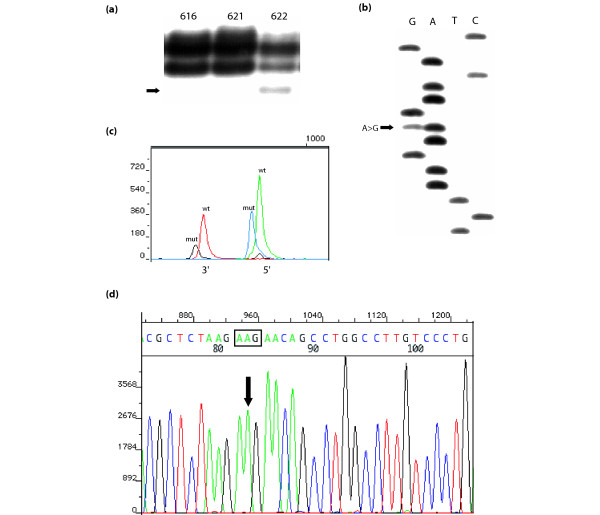
Comparison of screening methods in one ER-α A908G mutation-positive breast tumor from the CBCS. **(a) **Single-strand conformational polymorphism analysis of three Carolina Breast Cancer Study (CBCS) breast tumors, with the mutant band in tumor 622 indicated by an arrow. Tumors 615 and 617 contain only wild-type estrogen receptor (ER). **(b) **^33^P-cycle sequencing showing the A908G point mutation in CBCS breast tumor 622 (indicated by the arrow). **(c) **SNaPshot dideoxy primer extension analysis showing the wild-type (wt) and mutant (mut) ER peaks in both the 5' and 3' directions in CBCS breast tumor 622. Note that in the 5' direction the wild-type peak (A) is green and the mutant (G) is blue, whereas in the 3' direction, the wild-type peak (T) is red and the mutant (C) is black. **(d) **Big Dye terminator cycle sequencing analysis in the exon 4 region of ER-α surrounding codon 303 in CBCS breast tumor 622. Codon 303 is boxed; note that only the wild-type sequence (AAG) is observed.

We selected a panel of 10 CBCS breast tumors, 8 positive and 2 negative for the ER-α A908G mutation according to ^33^P-cycle sequencing (Table 2). The proportion of mutant in the eight positive tumors ranged from about 5% to 100% of template, on the basis of the relative intensities of mutant to wild-type bands on ^33^P-cycle sequencing autoradiographs. Fluorescent sequencing was initially performed on the 104-bp ER-α exon 4 fragment that was used in SSCP and ^33^P-cycle sequencing, but this small amplicon did not yield satisfactory fluorescent sequencing data because of its high G/C content and the close proximity of primers to the codon of interest. Primers were redesigned to generate a larger 219-bp ER-α exon 4 PCR product, which yielded good quality fluorescent sequencing. However, all eight ^33^P-cycle sequencing-positive tumors were completely negative by fluorescent sequencing, even though cloned DNA from the positive control breast tissue, which contained only mutant and no competing wild-type sequence, did exhibit the ER-α A908G mutation by this method. The SNaPshot method detected the mutation in seven of the eight positive tumors, but failed to identify it in one tumor in which it was present at less than 20% of total DNA template. An example of the results obtained using multiple screening methods for one ER-α A908G mutation-positive CBCS tumor is given in Fig. 2.

For further assessment of the relative sensitivities of the methods that successfully detected the ER-α mutation, several additional comparisons were made. ^35^S manual sequencing using the Sequenase version 2.0 Sanger method was performed on 20 CBCS breast tumors screened by ^33^P-cycle sequencing; of these, 11 were positive and 6 negative for the A908G mutation, and 3 were positive for the codon 311 variant. ^35^S manual sequencing produced the same results as ^33^P-cycle sequencing in all 20 samples.

The SNaPshot analysis was expanded to a total of 101 breast tumors (including the 10 in Table 2), 33 mutation-positive and 68 mutation-negative by ^33^P-cycle sequencing. Of the 33 A908G-positive tumors, 27 (82%) were also positive by SNaPshot, and these were all judged to contain more than 20% mutant template. The six SNaPshot-negative but ^33^P-cycle sequencing-positive tumors were estimated to contain 5 to 20% mutant. Thus, SNaPshot generally confirmed our findings with ^33^P-cycle sequencing when the proportion of mutant sequence in DNA samples was above 20%. We attempted to enhance the sensitivity of SNaPshot to detect the mutant by first digesting ER-α exon 4 with the *Mbo*II restriction enzyme, which cleaves only the wild-type sequence. One previous study used this approach in combination with fluorescent sequencing [20]. We found that digestion of ER-α exon 4 with *Mbo*II, followed by PCR re-amplification and SNaPshot analysis, led to a high frequency of false positive results for the ER-α A908G mutation, including positive findings in cell lines that had already been determined by ^33^P-cycle sequencing to contain only wild-type ER-α exon 4 (results not shown).

## Discussion

Our results confirm the presence of the ER-α A908G mutation in invasive breast cancer, although the overall mutation frequency is low. This finding is consistent with the literature, which indicates that mutations of the ER-α gene occur at low frequency in primary breast tumors [2,3]. The A908G base change was not detected in the germline of any case; this is consistent with the study of Fuqua and colleagues [9], which failed to find the mutation in normal breast epithelium adjacent to A908G mutation-positive breast hyperplasias. Schubert and colleagues [16] also evaluated the ER-α A908G base change as a potential polymorphic variant in the CBCS but failed to detect it in germline DNA.

Despite the initial finding of this mutation in hyperplastic breast lesions [9] and subsequently its detection in a high proportion of breast cancers [10], four recent studies by other researchers have failed to find the A908G mutation in either benign or malignant breast tissues [17-20]. It seems likely that multiple factors have contributed to the variable results across studies. These include laboratory screening methods, different tumor or patient characteristics of the populations evaluated, relatively small numbers of breast tumors evaluated previously, the apparent low prevalence of the mutation, its presence in a minority of cells within some tumor tissues, and a histologic preference for mixed lobular/ductal carcinomas that constituted fewer than 10% of breast tumors in the CBCS.

The previous negative studies evaluated primarily ductal carcinomas and included few, if any, mixed lobular/ductal carcinomas [17-20]. Two of the negative studies screened Japanese breast cancer patients who may have experienced different hormonal exposures from cases in our study [17,18]. One recent negative study assessed a highly selected group of ER expression-positive breast tumors from postmenopausal cancer patients treated with anti-estrogen therapy [20]. Our study, in contrast, assessed a large population-based series of more than 650 breast tumors of various histologic types from both premenopausal and postmenopausal women.

Laboratory screening techniques might have significantly contributed to the negative findings of several previous studies. One negative study relied on restriction digestion with the *Mbo*II restriction enzyme to detect the point mutation [17]. Detection of restriction products on agarose gels is relatively insensitive in comparison with radiolabeling techniques, because small amounts of undigested template, which could correspond to mutant, might not be visible, and incomplete digestion of wild-type template could be mistaken for mutant. In our hands, *Mbo*II was an inefficient cutter that never digested wild-type ER sequence to completion. This enzyme can also exhibit star activity, or inappropriate cutting, when digestion is allowed to proceed for more than the recommended time. Incomplete or inappropriate digestion by *Mbo*II may have contributed to background bands that we observed in sequencing and the false positive findings we obtained with the combination of *Mbo*II pre-digestion followed by SNaPshot. Similar anomalies associated with use of *Mbo*II were also reported recently by Davies and colleagues [20].

Three negative studies used automated fluorescent sequencing to screen for the A908G mutation [18-20]. Fluorescent sequencing has been reported to be less sensitive than radiolabeled sequencing for detecting somatic mutations, failing to detect one-third of mutations in the *p53* gene [21]. This inferior sensitivity has been attributed to unevenness in peak heights and suppression of peaks, particularly G when it follows A, resulting in inaccuracies in base calling [22,23]. Although the use of Big Dye terminators has at least partly corrected the differences in peak heights [22,23], we still observed a fourfold to fivefold variation in peak sizes within the ER exon 4 sequence and diminished G bases following A bases within the codon 302 to 303 sequence (AAG-AAG) using this method. The sensitivity of detection of the mutant G base in codon 303 (A**A**G to A**G**G) might be particularly low when it is present in only a small proportion of tumor cells.

Since the initial report by Fuqua and colleagues [9], we are the only researchers to have used radiolabeled techniques to detect the ER-α A908G mutation in breast tissues. The combination of SSCP and ^33^P-cycle sequencing proved to be a sensitive and reliable approach because SSCP demonstrated a consistent band shift pattern when the mutation was present, and even faint bands at the correct position on SSCP gels were usually indicative of the mutation. ^33^P-cycle sequencing, which permits more uniform labeling of all bases in a sequence, clearly detected the mutation, whereas fluorescent sequencing did not. It should be noted that ^35^S sequencing and SNaPshot dideoxy primer extension supported our findings with ^33^P-cycle sequencing.

The increased responsiveness of the mutant receptor to sub-physiologic levels of estrogen, and its detection in breast hyperplasias and now invasive carcinomas, raise the question of its role in breast cancer etiology and prognosis. The mechanism of mutant hypersensitivity is not an altered affinity for estradiol, because the mutant receptor binds hormone with affinity similar to that of wild-type ER-α [9]. Rather, it seems to be related primarily to an enhancement of phosphorylation at the downstream serine 305 residue via multiple kinase signaling pathways [10-12]. Thus, the mutation could function in the early stages of neoplastic development by stimulating cellular proliferation, increasing the formation of genetic changes associated with breast tumorigenesis. Our finding of higher tumor grade among the A908G mutation-positive tumors suggests that upregulated and/or aberrant estrogen signaling might be associated with the mutation and could lead to more aggressive tumor growth. It will be of interest to determine whether oral contraceptives, hormone replacement therapy or other endogenous hormonal factors interact with the ER-α A908G mutant to augment the growth of preneoplastic cells or established tumors. From the standpoint of therapy, it will also be important to determine whether the mutation influences response or the development of resistance to tamoxifen or other anti-estrogen therapies. A recent report by Michalides and colleagues [24] found that phosphorylation of serine 305 by PKA induces resistance to tamoxifen and may even convert tamoxifen from an antagonist to an agonist, although Fuqua and colleagues [9] did not find evidence for this in studies *in vitro*.

Our data from the CBCS indicate that the ER-α A908G mutation is present at a low frequency in invasive breast tumors and may occur more frequently in higher-grade cancers. The mutation may be associated with the mixed lobular/ductal tumor type, a less-characterized histologic entity, although this result was not statistically significant. Although the relationship of the hypersensitive ER-α A908G mutation to clinical and tumor growth characteristics is of significant interest, our results are based on a relatively small number of mutation-positive tumors even though the CBCS is the largest series of breast cancers yet screened for this mutation. Clearly, additional larger studies are needed to clarify the role of this mutant in breast cancer development.

## Conclusion

Our results confirm the presence of the ER-α A908G mutation, originally described in hyperplastic benign breast tissues, in some invasive breast cancers. Although the frequency of this mutation was low, it was more prevalent in subgroups of higher-grade breast tumors and those with mixed lobular/ductal histology. The presence of the hypersensitive ER-α A908G point mutation in invasive breast tumors may have important implications for breast cancer etiology and prognosis.

## Abbreviations

bp = base pair; CBCS = Carolina Breast Cancer Study; CI = confidence interval; ER-α = estrogen receptor-α ; OR = odds ratio; PCR = polymerase chain reaction; SSCP = single-strand conformational polymorphism; UNC = University of North Carolina.

## Competing interests

The authors declare that they have no competing interests.

## Authors' contributions

EP, DT and SNE conducted the laboratory analyses, JG, CL and HS conducted the pathology review, C-KT conducted the statistical analyses, and KC, RM, BN and SNE participated in the interpretation of results and writing of the manuscript. All authors read and approved the final manuscript.

## Figures and Tables

**Table 1:** Demographic and tumor characteristics of breast cancer cases overall and by ER-α A908G mutation status

Characteristic	Total cases (*N *= 653)	Mutation positive (*N *= 37)	Mutation negative (*N *= 616)	OR^a^	95% CI	*P*
	*n *(%)	*n *(%)	*n *(%)			
Age (years)						
50+	265 (40.6)	19 (51.4)	246 (40.0)	1.00		
<50	388 (59.4)	18 (48.6)	370 (60.0)	1.65	0.41–6.64	0.48
Race						
White	398 (60.9)	25 (67.6)	371 (60.2)	1.00		
African American	257 (39.1)	12 (32.4)	245 (39.8)	0.70	0.34–1.42	0.32
Menopausal status						
Postmenopausal	317 (48.5)	22 (59.5)	295 (47.9)	1.00		
Premenopausal	336 (51.5)	15 (40.5)	321 (52.1)	1.14	0.39–3.35	0.82
Stage^b^						
I	239 (39.3)	11 (35.5)	228 (39.5)	1.00		
II	298 (49.0)	16 (51.7)	282 (48.9)	1.35	0.61–3.00	0.46
III, IV	71 (11.7)	4 (12.8)	67 (11.6)	1.67	0.50–5.64	0.41
Primary tumor size						
≤ 2 cm	327 (52.3)	14 (42.4)	313 (52.9)	1.00		
>2 cm	298 (47.7)	19 (57.6)	279 (47.1)	1.81	0.87–3.74	0.11
Lymph node status						
Negative	377 (60.5)	18 (54.5)	359 (60.8)	1.00		
Positive	246 (39.5)	15 (45.5)	231 (39.2)	1.46	0.71–2.99	0.30
ER expression						
ER^+^	370 (58.8)	20 (58.8)	350 (58.8)	1.00		
ER^-^	259 (41.2)	14 (41.2)	245 (41.2)	1.24	0.60–2.56	0.57
Mitotic index^c^						
≤ 10	364 (56.4)	25 (67.6)	339 (55.8)	1.00		
>10	281 (43.6)	12 (32.4)	269 (44.2)	0.74	0.35–1.54	0.42
Histologic grade						
Well to moderately differentiated	221 (34.1)	11 (29.7)	210 (34.3)	1.00		
Poorly differentiated	428 (65.9)	26 (70.3)	402 (65.7)	1.43	0.69–2.99	0.34
Nuclear grade						
Slight to moderate pleomorphism	375 (57.7)	20 (54.1)	355 (57.9)	1.00		
Marked pleomorphism	275 (42.3)	17 (45.9)	258 (42.1)	1.48	0.74–2.97	0.27
Combined tumor grade^d^						
I	162 (25.2)	6 (16.2)	156 (25.7)	1.00		
II	198 (30.7)	18 (48.7)	180 (29.7)	2.83	1.09–7.34	0.03
III	284 (44.1)	13 (35.1)	271 (44.6)	1.65	0.60–4.59	0.33
Histologic type(s)						
Ductal NOS^e^	513 (78.6)	27 (73.0)	486 (78.9)	1.00		
Ductal variants^f^	21 (3.2)	1 (2.7)	20 (3.2)	0.69	0.09–5.42	0.72
Lobular^g^	58 (8.9)	2 (5.4)	56 (9.1)	0.57	0.13–2.50	0.46
Mixed lobular/ductal	61 (9.3)	7 (18.9)	54 (8.8)	2.10	0.86–5.12	0.10

^a^Odds ratios adjusted for age and race. ^b^According to the American Joint Committee on Cancer breast tumor staging guidelines. ^c^Per 10 high power fields. ^d^Composite score based on mitotic index, histologic grade and nuclear grade. ^e^Ductal NOS (not otherwise specified), *n *= 462; medullary, *n *= 4; neuroendocrine, *n *= 2; poorly differentiated, *n *= 13; metaplastic, *n *= 5; other mixed, *n *= 27. ^f^Ductal variants include mucinous (*n *= 11), tubular (*n *= 2), papillary (*n *= 4), and other (*n *= 4). ^g^Lobular, classic and/or variant. CI, confidence interval; ER, estrogen receptor.

**Table 2:** Comparative results of screening methods to detect the ER-α A908G mutation

CBCS tumor	^33^P-cycle sequencing	Fluorescent sequencing	SNaPshot primer extension
28	+	-	+
36	+	-	+^b^
313	+	-	+
536	+	-	+
622	+^a^	-	+
684	+	-	+
705	+	-	+^b^
710	+^a^	-	-
162	-	-	-
297	-	-	-
Positive control tissue	+	-	+
Mutant clone from positive control	+	+	n.d.

^a^The mutation was also confirmed by ^35^S manual sequencing in these samples; other tumors shown were not evaluated by this method.

^b^Mutant peaks were small, indicating low-level positivity.

CBCS, Carolina Breast Cancer Study; ER, estrogen receptor; n.d., not determined.
